# Riverine fishers’ knowledge of extreme climatic events in the Brazilian Amazonia

**DOI:** 10.1186/s13002-016-0123-x

**Published:** 2016-10-26

**Authors:** Ana Isabel Camacho Guerreiro, Richard J. Ladle, Vandick da Silva Batista

**Affiliations:** 1 National Institute of Amazonian Research (INPA), Av. André Araújo, 2.936, Petrópolis. CEP 69.067.375, Manaus, AM Brazil; 2 Federal University of Alagoas (UFAL), Institute of Biological and Health Sciences (ICBS), Campus A. C. Simões; Av. Lourival Melo Mota s/n, Tabuleiro dos Martins. CEP 57.072.900, Maceió, AL Brazil; 3 School of Geography and the Environment, University of Oxford, Oxford, UK

**Keywords:** Ethnoclimatology, Historical knowledge, Climate change impacts, Cultural memory, Fishery resources, Etnoclimatologia, Conhecimento histórico, Impactos da mudança climática, Memória cultural, Recursos pesqueiros

## Abstract

**Background:**

Climate change is altering climate patterns, mainly increasing the frequency and intensity of extreme events with potentially serious impacts on natural resources and the people that use them. Adapting to such impacts will require the integration of scientific and local (folk) knowledge, especially the first-hand experiences and perceptions of resource users such as fishers. In this study, we identify how commercial riverine fishers in the Amazon remember extreme climatic events (flood and drought) and how they face the consequences of extreme events on fish availability.

**Methods:**

Data were collected from the main Manaus fishery harbor between June and October of 2013. Semi-structured questionnaires and a historical timeline technique were used to gather data from artisanal commercial fishers. Fishers’ knowledge of extreme climate events was assessed by their “cultural consensus” for identification of event years and perceived impacts. Fishers’ responses were also compared to hydrological data to test their similarity.

**Results:**

There was a high level of cultural consensus among fishers about extreme events years. They were able to identify four consecutive unusual droughts, between 2009 and 2012. Elevated levels of fish mortality and decreases in the fishery were perceived as consequences of the drought events, as well as, a reduction in fish size, and disappearance of some species. Extreme flood events were associated with greater difficulties accessing fishing grounds.

**Conclusions:**

Extreme climatic events (floods and droughts) were remembered, and the recent increase in their intensity and frequency was also perceived. Moreover, extreme climate event (mainly droughts) impacts on fishery resources were also observed. Such information is potentially valuable for educational programs to further improve adaptation of local Amazonian fishing communities to future climate change, e.g. increasing local ecological knowledge using learning material based on their perception.

## Resumo

**Contexto:**

A mudança climática está alterando os padrões climáticos, por meio de um aumento tanto da frequência como da intensidade de eventos climáticos extremos com potenciais impactos nos recursos naturais e nos seus utilizadores. A adaptação a tais impactos exigirá uma integração entre os conhecimentos científico e local, principalmente as experiências diretas e percepções de utilizadores dos recursos tais como os pescadores. No presente estudo, identificámos como os pescadores comerciais ribeirinhos da Amazônia recordam os eventos climáticos extremos (cheia e seca) e como estes enfrentam as consequências dos eventos extremos na disponibilidade de peixe.

**Métodos:**

Os dados foram coletados no principal porto de pesca de Manaus entre os meses de junho e outubro de 2013. Os dados foram coletados de pescadores artesanais comerciais por meio de entrevistas semi-estruturadas e por meio da técnica “linha cronológica histórica”. O conhecimento que estes pescadores tinham sobre eventos climáticos extremos foi avaliado por meio do “consenso cultural” na identificação de anos de eventos extremos e dos impactos perceptionados por estes pescadores. Além disso, as respostas obtidas foram também comparadas com dados hidrológicos de forma a testar a sua similaridade.

**Resultados:**

O nível de consenso cultural acerca de anos de eventos extremos foi elevado entre os pescadores. Eles foram capazes de identificar quatro anos consecutivos de seca excepcional, registados entre 2009 e 2012. Níveis elevados de mortalidade de peixe e diminuição na pesca foram percepcionados como consequências de eventos de seca, bem como, a diminuição no tamanho do peixe e o desaparecimento de algumas espécies. Os eventos de cheia extrema foram associados a maiores dificuldades de acesso aos bancos de pesca.

**Conclusões:**

Os eventos climáticos extremos (cheias e secas) foram recordados, bem como o recente aumento na intensidade e frequência destes extremos. Adicionalmente, os impactos de eventos climáticos extremos (principalmente secas) nos recursos pesqueiros também foram observados. Esta informação é potencialmente útil em programas educacionais para que comunidades pesqueiras locais da Amazônia possam à posteriori melhor se adaptar ás mudanças climáticas futuras, por exemplo, por meio do aumento de material educativo acerda de conhecimento ecológico local baseado nas suas percepções.

## Background

Climate change is significantly altering the structure and function of ecosystems, compromising their ability to provide goods and services to human populations [1, 2]. Some projections indicate that water availability, thermal regimes and biogeochemical processes will be strongly affected in some geographic regions [3]. Consequently, freshwater biodiversity, the structure and dynamic of the aquatic food webs, as well as the quality, abundance and distribution of habitats will be altered [3]. Freshwater fisheries could also be affected by changes in water temperature that will influence habitat quality, and by changes in water volume that will affect habitat quality and availability, habitat connectivity, and lifecycle events [4–7]. This will happen while freshwater fishes are threatened by other anthropogenic impacts, including the degradation of the riparian habitat, imposition of migration barriers, the introduction of exotic species [8], and overfishing [9]. Such impacts are global and are even apparent in the largest rivers of the world such as the Amazon [10]. Finally, environmental change and globalization are impacting social-ecological systems, as the collapse of fish stocks can lead to social reorganization [11].

The interaction and connection between ecological and social subsystems is an important mediator of the influence of environmental change on natural resources. In fisheries context, the fishers’ ecological knowledge (EK) represents a substantial information base and is frequently used to evaluate fisheries resource levels [11–14], plan fisheries conservation [15–19], and to support basic research on local fish populations [18]. Such knowledge is increasingly being applied to the study of climate change through ethno-climatological approaches [20–22], which can be utilized to determine the potential consequences of climate change on exploited populations across different spatial and temporal scales. Information from such studies can be used for participative management [23–25] and to guide future research [26]. Moreover, the integration of scientific and traditional knowledge facilitates and supports social negotiation during the development and implementation of resource management measures [15, 27–29].

Artisanal fishing communities in the Amazon have historically been subjected to the impacts of climatic variabilities, such as El-Niño and La Niña and they have developed strategies to deal with extreme phenomena. However, new challenges in the form of current climate change may affect their economy and food security [1]. Climate change may, therefore, increase fishers’ dependence on having sound knowledge of climate and its effects to develop effective decision-making. In Brazil, studies on ethnoichthyology have mostly been applied in coastal areas with less attention given to the large fishing communities of the Amazon [30], characterized by high levels of subsistence consumption [31] and commercial activity [32].

The aims of this study were to: 1) assess how commercial riverine fishers recall extreme climatic events affecting the local aquatic environments (drought and flood); 2) identify how fishers respond to the consequences of extreme events on fish availability; 3) determine the sources of information about extreme events (drought and flood) used by fishers, considering their education and experience, and; 4) assess fishers’ knowledge of extreme event years. To do that, we adopted two approaches: 1) Cultural response analysis based on “cultural consensus theory”; 2) integrative fishers/hydrological analysis: comparing empirical measurements of water levels with fishers’ identification of years of extreme events.

## Methods

### Study area

The Amazon region is made up of vast areas of primary and secondary forest, and a few (mainly) small (<50 thousand inhabitants) urbanized areas with a racially mixed population. Several tribal groups—most already acculturated, but others still non-contacted—inhabit the region, creating complex social-biological diversity. As the rivers remain the main communication pathways in the area, hydrological fluctuations strongly modulate the social-cultural dynamics [33].

Amazonian forests significantly contribute to the regional and global regulation of climate [34], being important in a climate change context. The climate of Central Amazon is hot and humid, with a mean annual temperature of 26.6 °C. During the dry period, evaporation can surpass precipitation [35, 36]. During the warm ENSO-phases (El Niño) flood levels are typically lowered, and aquatic phases are shortened, while high and prolonged flooding is associated with cold ENSO-phases (La Niña) [37].

### Data collection

**Fig. 1:**
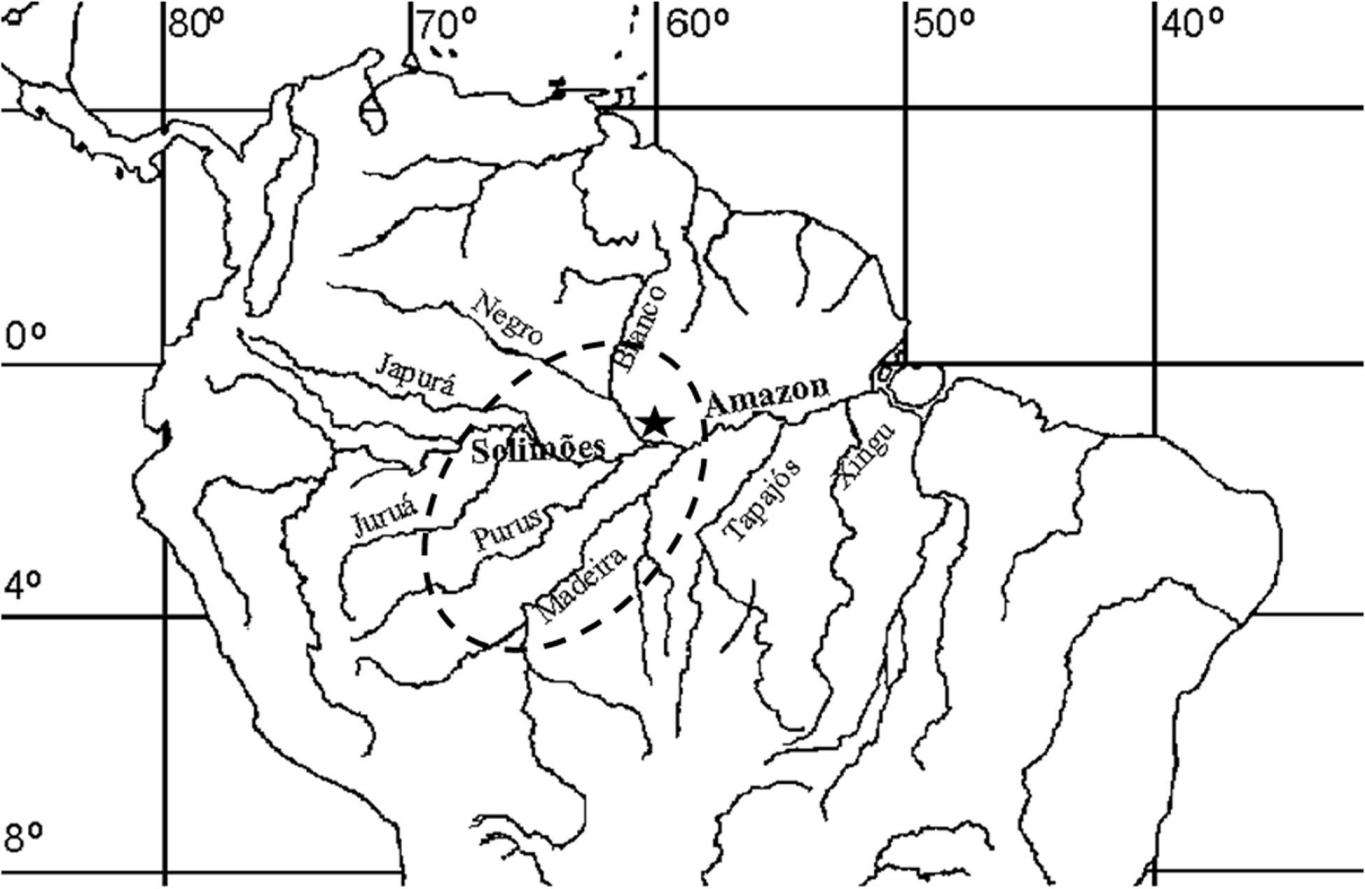
Map of Central Amazonia. Legend: *Black Star* - Panair’ fishery harbor, located in Manaus

Data was collected between June and October 2013 through interviews with commercial fishers that use the Panair fishery harbor in Manaus, Central Amazon (Fig. 1). This is the main fishery landing and commercial center of central Amazonia, receiving 25 % of the fish *in nature* commercialized in the Region [38].

Semi-structured questionnaires (see Appendix) were applied to 91 artisanal commercial fishers. A stratified random sample was applied by separating the population into strata based on fishermen’s experience (“novice fishers”- less than 15 years of experience; and “experienced fishers”- fishers with 15 plus years of experience) to assure the representativeness of the sample and the perception of the error [39]. The interviews followed standard recommendations for ethnoecological research [39]. They lasted for about 30 min and were given in Portuguese. Participant observation and historical timelines were applied to the questionnaires [39]. Questions dealt with various social and cultural themes, e.g., time of fishery experience, education, sources of information and ecological knowledge related to fish, fishing and climate, including perceived impacts of extreme weather impacts on local fishery resources. All research was conformed to the ethical and legal obligations of the author’s institution and the Federal government (license number: 13813613.9.0000.0006).

### Data analysis

Sources of knowledge of extreme climatic events were classified as familiar (grandparents, parents, brothers and cousins) and non-familiar (magazines and personal experience). Differences between sources of knowledge for each experience category (experienced/novice) were assessed. Fishers’ educational level was classified as literate (basic, primary/new fundamental education) or illiterate (“did not study”). Differences between educational level and Fisher categories (experienced/novice) were assessed.

Two methodologies were used to evaluate the fishers’ knowledge of past extreme climate events: a consensus analysis and an integrative fishers/meteorological analysis. The consensus analysis [40, 41] was performed and validated using anthropological theory [42]. In this approach, the cultural consensus is defined as the “pattern or agreement or consensus among informants that permit to make inferences about their differential competence in knowledge of the shared information pool constituting the culture. It is assumed that the correspondence between the answers of any two informants is a function of the extent to which each is correlated with the truth” [40]. To support this analysis we estimated and plotted the annual distribution frequency of quotes by drought events and by flood events.

The integrative fishers/meteorological analysis was based on hydrological records of water levels were retrieved from the Manaus harbour website [43]. Extreme flood/drought events were classified as those causing a water level of above 28 m or lower than 17 m, respectively, according to the criteria of Bittencourt and Amadio [44]. Fishers’ memories of extreme events were classified as being “in Accordance with the Hydrological Records” (AHR) or “Not in Accordance with the Hydrological Records” (NAHR).

### Statistical analysis

A Pearson’ chi-squared test with Yates’ continuity correction was used to compare fishers’ experience categories with 1) sources of extreme events knowledge (Familiar/Not Familiar) and with 2) education levels (Literate/Illiterate).

A Pearson’ chi-squared test with Yates’ continuity correction was also used to test differences in AHR between experience categories (experienced/novice) and between fishing grounds (one or more than one fishing ground) for each of the extreme events. Finally, differences in AHR between types of event (flood/drought) were also assessed. All statistics were performed in the *R* software environment [45], and all the statistical tests were at the 5 % significance level.

## Results

### Social survey

**Fig. 2:**
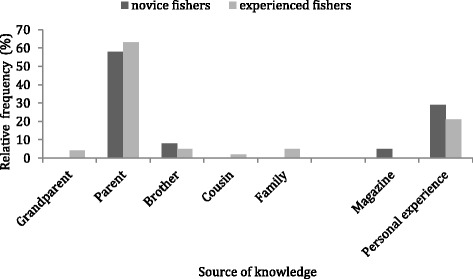
Sources of information (%) (24 quotes - experienced fishers; and 56 quotes - novice fishers) (Familiar sources: grandparent, parent, brother, cousin and family; not familiar sources: magazine and personal experience)

**Fig. 3:**
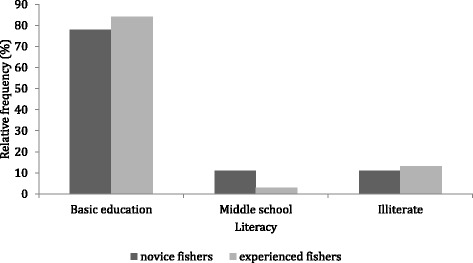
Fishers’ educational level (basic education: old primary school and modern fundamental education; middle school; and illiteracy) (36 novice fishers and 55 experienced fishers)

The information sources for knowledge of extreme events were similar for experienced and inexperienced fishers (*P* = 0.08). Even so, differences in familiar knowledge acquisition were found as we have only noticed familiar restricted sources (brother, parent, grandparent, cousin) for old fishers and familiar nuclear sources (parent and brother) for novice fishers (Fig. 2). No significant association was found between educational level and experience category (*P* = 0.83) (Fig. 3).

### Extreme climatic knowledge

**Fig. 4:**
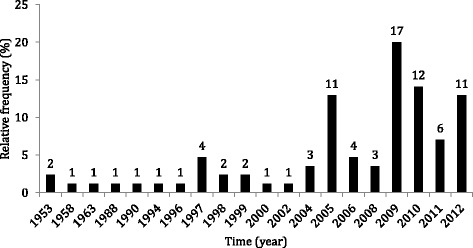
Fishers’ identification of years of flood events (%) (81 quotes)

**Fig. 5:**
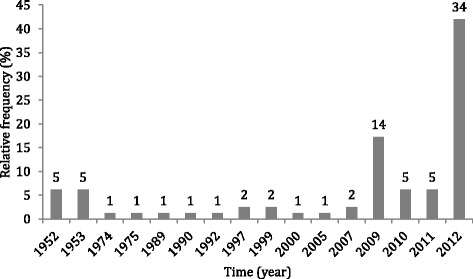
Fishers’ identification of years of drought events (%) (85 quotes)

Fishers were relatively proficient at identifying the dates of extreme climatic events, whether assessed by cultural response consensus or integrative fishers/meteorological analysis. According to the consensus analysis, interviewers identified 16 different years with extreme flood events. Additionally, the four most cited years accounted for 71 % of all responses - the flood of 2012 was cited by 42 % of interviewees, and the floods of 1953, 2009, and 2011 were recognized by 29 % of the fishers (Fig. 4). Similarly, 20 different years were identified as those remarkable for extreme drought events, the seven most cited accounting for 77 % of the quoted events (Fig. 5), including four consecutive unusual droughts (2009–2012). Moreover, the proportion of answers that were concordant with the hydrological records were similar for flood and drought events (*P* = 0.28). Fishers that fish in one or more than one fishing ground also provided similar responses (*P* = 0.11). Furthermore, many of the fishers interviewed mentioned that droughts and floods are becoming more intense and more frequent, indicating that they may be perceiving the influence of anthropogenic climate change.

**Fig. 6:**
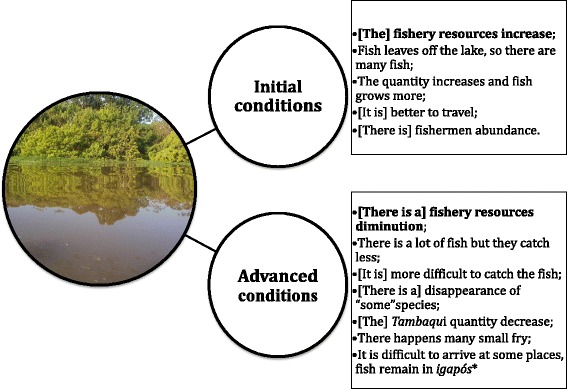
Extreme flood impacts as perceived by the fishers (translated quotes)

**Fig. 7:**
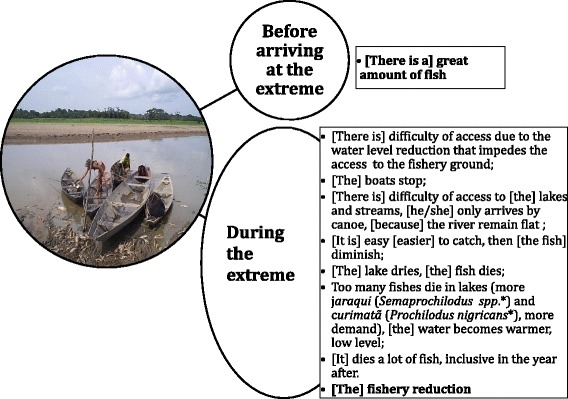
Extreme drought impacts as perceived by the fishers (translated quotes)

Perceptions of extreme climatic impacts were grouped according to the ecological conditions that occur during this type of event (Figs. 6 and 7). Fishers were more able to identify the impacts of extreme droughts (73 %) than floods (37 %). Extreme flood events are considered favorable in the initial phase, as fish growth is thought to be higher than in advanced conditions of a flood. Fishers aggregate at the river and lakes mouths in the early phases of the flood. However, as the flood event advances this scenario changes because some species disappear, and other commercially important fishes such as the black pacu (*Colossoma macropomum*) decrease. Finally, fishers also have difficulty in accessing the *igapós* (flood forest) where fish remains (Fig. 6).

At the beginning of drought events, fishers described that they typically have access to large quantities of fish as ecologically appropriate decrease in area. However, further reduction in the water level reduces the connectivity of the lakes, forcing fishers to carry canoes by hand and reducing the total catch. As the drought advances, there is also a significant increase of commercially valuable fish in lakes, mainly the silver and flagtail prochilodus (*Semaprochilodus* spp.) and black prochilodus (*Prochilodus nigricans*). Close to the minimum water level, fish are no longer available for fishers due to lack of access or complete loss of fish stocks. Fishers were also able to detect a delayed response regarding fish mortality (Fig. 7).

## Discussion

Artisanal fishers are clearly very knowledgeable about extreme climatic events in the past. Both the cultural consensus and complementary approaches were similarly effective in identifying past extreme events. Furthermore, fishers had detailed perception about drought impacts on fisheries resources. In South America, research has only been conducted on fishers’ perceptions of birds biology [46], the interaction between birds and fishing [47], and territorial use rights [48]. However, the study contributes to the global literature of fishers’ perceptions of environmental change [49–52], and similar results were recorded by West and Vásquez-León [53] and Puri [54] for farmer’s perception of meteorological records.

Evidence was found that fishers with different levels of experience use different knowledge sources. However, fishers’ experience, the number of fishing grounds and educational level did not affect their recall. Such results are concordant with the historical dynamics of environmental, social and economic conditions in Central Amazon. An integrated environmental-economic example is the implementation and maintenance of new fishing technologies and the spread of commercial-scale fishing during the 1960s [55, 56]. Rapid social change and intergenerational differences in knowledge, as reported in this study, are common phenomena in subsistence cultures [57]. However, the adaptive character of local knowledge allows effective responses to changing conditions [12, 58]. This is broadly supported by our results since fishers appear to be using different knowledge sources to identify the extreme events and to make inferences about them.

Fishers had the capacity to identify the different impacts that extreme droughts and floods had on fishing resources. Specifically, they identified an increase in fish abundance during the initial phase of extreme droughts—as during ‘normal’ drought conditions. However, during peak periods of extreme droughts they perceived fish mortality to be unusually high (and varied between species) and a reduction in fish quality. From the fishers’ perspective, these events are memorable since they necessitate considerable changes in fishing behavior as access to common fishing grounds become harder.

Fishers’ observations effects of drought on fishes in our study are concordant with scientific data from the USA [59], which identifies factors such as 1) fish population decline; 2) loss of habitats, and; 3) agglomeration of fish. More generally, adverse effects of droughts on food production have been reported by Nakashima et al. [60] for various crops, who note that local communities believe that “ENSO- related period of drought tend to be related with little to no production of ceremonial yams and kava, while wetter years produce the contrary effect”. Although too obvious, this is an example how traditional knowledge of local people links natural resource fluctuations to climatic events.

The recent increase in severity and frequency of extreme climatic events [61, 62] was clearly perceived by the fishers. Such sensitivity to subtle changes in climate has been reported as contributing to the better understanding of the local expressions of global climate change [63]. Taking into consideration that “memory” is a cognitive process [20] - understood in anthropological studies as a valuable tool to build system resilience [64] - those eventual, but remarkable, events are key for strengthening communities to react to future increases in climatic extremes. Furthermore, the capacity to identify [65] and anticipate changes in future weather conditions is one of best adaptation measures to climate changes [66, 67], addressing the dual challenge of resource conservation and poverty alleviation [68].

Resource users knowledge of past climatic events is a potentially valuable source of information on the intensity and timing of climate change and can provide additional information that has not been recorded by scientific research [22, 67]. More detailed information to increase our understanding of spatial-temporal climatic-resource dynamics in the region could be gained through participatory mapping, widely used in studies of environmental conservation, cultural preservation, and climate change adaptation [69]. Furthermore, since local ecological knowledge was a strong source for the identification of extreme events, the development of educational programs are recommended to increase the local fishing communities’ resilience facing climate change notwithstanding other social benefits from education. Learning material explicitly based on cultural perceptions can motivate technological/behavioral adaptations and innovations, strengthening fishers’ power during negotiations with the representatives of government agencies [22, 63].

## Conclusions

Artisanal commercial fishers in the Amazon recall extreme events. They are also able to identify the main impacts of extreme climatic events on the fisheries resources they exploit. Such knowledge is clearly useful for building consensus on adaptation strategies and, potentially, for introducing measures to promote sustainable fisheries during times of climate change. Moreover, fishers appear to be valuable repositories of climate memory at a local scale, a characteristic that could be exploited to deal with challenges related to climatic change.

Despite the promising results of this study, further research is needed that incorporates other aspects of ethno-climatological memory analysis, such as cultural values and frames, long-term memory and short-memory characteristics and/or the level of externally-built awareness of climate change. This is the key to bottom-up approaches of social development using natural resources.

## Appendix

### Supplementary Material

#### Translated questionnaire questions

- Date:
- Questionnaire number:
  - 1) Are you commercial fishermen? [If response is no] What type of fishermen do you are?
  - 2) How many years were you fishing?
  - 3) How many years did you study?
  - 4) Who have taught you about fisheries?
  - 5) Where do you use to fish?
  - 6) Do you know that great floods and droughts that used to happen here in the Amazonia?
    - 6.1.) What are the main changes that you have noted in fishery resources during great droughts? What about during great floods?
  - 7) Do you remember any year of great drought? What about a great flood?

## References

[1] IPCC: Summary for Policymakers. In: Climate Change 2013: The Physical Science Basis. Contribution of Working Group I to the Fifth Assessment Report of the Intergovernmental Panel on Climate Change. 2013.

[2] Hurrell JW, Deser C. North Atlantic climate variability: The role of the North Atlantic Oscillation. Journal of Marine Systems. 2010;79:231–244.

[3] Wrona FJ, Prowse TD, Reist JD, Hobbie JE, Lévesque LMJ, Vincent WF (2006). Climate change effects on aquatic biota, ecosystem structure and function. Ambio.

[4] Feenstra JF, Burton I, Smith JB, Tol RSJ. Handbook on Methods for Climate Change Impact Assessment and Adaptation Strategies. The Netherlands: UNEP;1998.

[5] Nolan KS, Fabré NN, Batista VS (2009). Landscape variables affecting fishery yield in lake systems of the Central Amazon region, Brazil. J Appl Ichthyol.

[6] Stassen MJM, van de Ven MWPM, van der Heide T, Hiza MAG, van der Velde G, Smolders AJP (2010). Population dynamics of the migratory fish Prochilodus lineatus in a neotropical river: the relationships with river discharge, flood pulse, El Niño and fluvial megafan behaviour. Neotrop Ichthyol.

[7] Siegel DA, Mitarai S, Costello CJ, Gaines SD, Kendall BE, Warner RR (2008). The stochastic nature of larval connectivity among nearshore marine populations. Proc Natl Acad Sci U S A.

[8] Cooke S, Paukert C, Hogan Z (2012). Endangered river fish: factors hindering conservation and restoration. Endanger Species Res.

[9] Welcomme RL, Cowx IG, Coates D, Bene C, Funge-Smith S, Halls A (2010). Inland capture fisheries. Philos Trans R Soc B, Biol Sci.

[10] Castello L, Mcgrath DG, Hess LL, Coe MT, Lefebvre PA, Petry P (2013). The vulnerability of Amazon freshwater ecosystems. Conserv Lett.

[11] Perry RI, Ommer RE, Barange M, Jentoft S, Neis B, Sumaila UR: Marine social – ecological responses to environmental change and the impacts of globalization. Fish Fish 2011;12(4):1–24.

[12] Berkes F, Colding J, Folke C (2000). Rediscovery of traditional ecological knowledge as adaptive management. Ecol Appl.

[13] Gomez-Baggethun E, Reyes-Garcia V, Olsson P, Montes C (2012). Traditional ecological knowledge and community resilience to environmental extremes: A case study in Donana, SW Spain. Glob Environ Chang Policy Dimens.

[14] Begossi A, Caires R (2015). Art, fisheries and ethnobiology. J Ethnobiol Ethnomed.

[15] Shackeroff JM, Campbell LM (2007). Traditional Ecological Knowledge in Conservation Research : Problems and Prospects for their Constructive Engagement. Conserv Soc.

[16] Murray, C, Wieckowski, K, Hurlburt, D, Soto, C, Johnnie K. Incorporation of Traditional and Local Ecological Knowledge and Values in Fisheries Management. Canada: ESSA Technologies Ltd.; 2011.

[17] Begossi A, Figueiredo J (1995). Ethnoichtyology of southern coastal fishermen:cases from Buzios Island and Sepetiba Bay (Brazil). B Mar Sci.

[18] Santos CAB, Nóbrega Alves RR (2016). Ethnoichthyology of the indigenous Truká people, Northeast Brazil. J Ethnobiol Ethnomed.

[19] Silvano RAM, Silva AL, Ceroni M, Begossi A (2008). Contributions of ethnobiology to the conservation of tropical rivers and streams. Aquat Conserv.

[20] D’Andrade RG: The Development of Cognitive Anthropology. Cambridge: Cambridge University Press; 1995.

[21] Flinn MV (1997). Culture and the evolution of social learning. Evol Hum Behav.

[22] Peterson N, Broad K: Climate and Weather Discourse in Anthropology: From Determinism to Uncertain Futures. In Anthropology and Climate Change: From Encounters to Actions. Edited by Crate S, Nutall M. Walnut Creek, CA: Left Coast Press; 2009:70–86.

[23] Mackinson S, Nøttestad L (1998). Combining local and scientific knowledge. Rev Fish Biol Fish.

[24] Hill NAO, Michael KP, Frazer A, Leslie S (2010). The utility and risk of local ecological knowledge in developing stakeholder driven fisheries management: The Foveaux Strait dredge oyster fishery, New Zealand. Ocean Coast Manag.

[25] Pinto MF, Mourão JS, Alves RRN (2015). Use of ichthyofauna by artisanal fishermen at two protected areas along the coast of Northeast Brazil. J Ethnobiol Ethnomed.

[26] Pinto M, Mourão J, Alves R (2013). Ethnotaxonomical considerations and usage of ichthyofauna in a fishing community in Ceará State, Northeast Brazil. J Ethnobiol Ethnomed.

[27] Moller H, Berkes F (2004). Combining science and traditional ecological knowledge: monitoring populations for co-management. Ecol Soc.

[28] Yli-Pelkonen V, Kohl J (2005). The role of local ecological knowledge in sustainable urban planning : perspectives from Finland. Sustain Pract Policy.

[29] Wilson DC, Raakjær J, Degnbol P (2006). Local ecological knowledge and practical fisheries management in the tropics: A policy brief. Mar Policy.

[30] Alves RR, Souto WM (2011). Ethnozoology in Brazil: current status and perspectives. J Ethnobiol Ethnomed.

[31] Batista VS, Inhamuns AJ, Freitas CEC, Freire-Brasil D (1998). Characterization of the fishery in river communities in the low-Solimoes/high-Amazon region. Fish Manag Ecol.

[32] Batista VS, Petrere M (2003). Characterization of the commercial fish production landed at Manaus, Amazonas State, Brazil. Acta Amaz.

[33] Schor T, Marinho RR, Costa DP, Oliveira JA (2014). Cities, Rivers and Urban network in the Brazilian Amazon. Brazilian Geogr J Geosci Humanit Res Mediu.

[34] Furley P: Tropical forest of the lowlands. In The physical geography of South America. seventh. Edited by Veblen T, Young K, Orme A. New York: Oxford University Press; 2007:361.

[35] Junk WJ (Ed): The Central Amazon Floodplain: Ecology of a Pulsing System. Berlin Heildelberg: Springer; 1997.

[36] Hodnett MG, Silva LP, Rocha HR, Cruz Senna R (1995). Seasonal soil water storage changes beneath central Amazonian rainforest and pasture. J Hydrol.

[37] Schöngart J, Junk WJ (2007). Forecasting the flood-pulse in Central Amazonia by ENSO-indices. J Hydrol.

[38] Parente VM, Batista VS (2005). A organização do desembarque e o comércio de pescado na década de 1990 em Manaus, Amazonas. Acta Amaz.

[39] Albuquerque UP, Cruz da Cunha LVF, Lucena RFP. Methods and Techniques Ethnobiology and Ethnoecology. New York: Springer Science + Business Media; 2014.

[40] Romney AK, Weller SC, Batchelder WH (1986). Culture as Consensus: A Theory of Culture and Informant Accuracy. Am Anthr.

[41] Cavalcanti IFA (2012). Large scale and synoptic features associated with extreme precipitation over South America: A review and case studies for the first decade of the 21st century. Atmos Res.

[42] Kroeber A: Anthropology: Race, Language, Culture, Psychology, Prehistory. New York; 1948.

[43] Level of the Negro River (Nível do Rio Negro)

[44] Bittencourt MM, Amadio SA (2007). Proposta para identificação rápida dos períodos hidrológicos em áreas de várzea do rio Solimões-Amazonas nas proximidades de Manaus. Acta Amaz.

[45] Dunn PK: tweedie: Tweedie exponential family models. R package version 1.5.2. 2007.

[46] Andrade LP, Silva-Andrade HML, Lyra-Neves RM, Albuquerque UP, Telino-Júnior WR (2016). Do artisanal fishers perceive declining migratory shorebird populations?. J Ethnobiol Ethnomed.

[47] Suazo CG, Schlatter RP, Arriagada AM, Cabezas LA, Ojeda J (2013). Fishermen’s perceptions of interactions between seabirds and artisanal fisheries in the Chonos archipelago, Chilean Patagonia. Oryx.

[48] Gelcich S, Cinner J, Donlan C, Tapia-Lewin S, Godoy N, Castilla J. Fishers’ perceptions on the Chilean coastal TURF system after two decades: problems, benefits, and emerging needs. Bull Mar Sci. 2016;92:000-000.

[49] Musinguzi L, Efitre J, Odongkara K, Ogutu-Ohwayo R, Muyodi F, Natugonza V (2016). Fishers’ perceptions of climate change, impacts on their livelihoods and adaptation strategies in environmental change hotspots: a case of Lake Wamala, Uganda. Environ Dev Sustain.

[50] Sievanen L (2014). How do small-scale fishers adapt to environmental variability? Lessons from Baja California, Sur, Mexico. Marit Stud.

[51] Conley KR, Sutherland KR (2015). Commercial fishers’ perceptions of jellyfish interference in the Northern California Current. ICES J Mar Sci.

[52] Coll M, Carreras M, Ciércoles C, Cornax MJ, Gorelli G, Morote E, Sáez R. Assessing fishing and marine biodiversity changes using fishers’ perceptions: The Spanish Mediterranean and Gulf of Cadiz case study. PLoS One. 2014;9:e85670. 10.1371/journal.pone.0085670 PMC3899065 24465644

[53] West C, Vásquez-León M: Testing farmers’ perceptions of climate variability:A case study from the Sulphur Sping Valley, Arizona. In Weather, climate and culture. Edited by Strauss S, Orlove B. Oxford: Berg; 2003:233–250.

[54] Puri R: Responses to medium-term stability in climate: El Niño, droughts, and coping mechanisms in foragers and farmers in Borneo. In Modern crises and traditional strategies: Local ecological knowledge in island southeast Asia. Edited by Ellen R. New York: Berghahn Books; 2007:46–83.

[55] Ferreira AR: Viagem Filosófica Pelas Capitanias Do Grão Pará, Rio Negro, Mato Grosso E Cuiabá (1792). Rio de Janeiro: Cons. Fed. de Cultura; 1974.

[56] Batista V, Petrere M (2007). Spatial and temporal distribution of fishing resources exploited by the Manaus fishing fleet, Amazonas, Brazil. Brazilian J Biol.

[57] Gómez-Baggethun E, Reyes-García V (2013). Reinterpreting Change in Traditional Ecological Knowledge. Hum Ecol.

[58] Reyes-García V, Luz AC, Gueze M, Paneque-Gálvez J, Macía MJ, Orta-Martínez M (2013). Secular trends on traditional ecological knowledge: An analysis of changes in different domains of knowledge among Tsimane’ men. Learn Individ Differ.

[59] Matthews WJ, Marsh-Matthews E (2003). Effects of drought on fish across axes of space, time and ecological complexity. Freshw Biol.

[60] Nakashima DJ, Galloway MK, Thulstrup HD CR& RJ: Weathering Uncertainty: Traditional Knowledge for Climate Change Assessment and Adaptation. Paris: UNESCO; 2012.

[61] Allison EH, Perry AL, Badjeck M-C, Neil Adger W, Brown K, Conway D (2009). Vulnerability of national economies to the impacts of climate change on fisheries. Fish Fish.

[62] Chambers JQ, Tribuzy ES, Toledo LC, Crispim BF, Higuchi N, Santos J, Araújo AC, Kruijt B, Nobre AD, Trumbore SE (2004). Respiration from a tropical forest ecosystem: partitioning of sources and low carbon use efficiency. Ecol Appl.

[63] Roncoli C, Crane T, Orlove B: Fielding Climate Change in Cultural Anthropology. In Anthropology and climate change : from encounters to actions. Edited by Crate AS, Nuttall M. Walnut Creek, CA: Left Coast Press; 2009:87–115.

[64] Berkes F, Colding J, Folke C (2003). Navigating Social-Ecological Systems: Building Resilience for Complexity and Change.

[65] Alves R, Souto W (2015). Ethnozoology: A Brief Introduction. Ethnobiol Conserv.

[66] Kolawole OD, Wolski P, Ngwenya B, Mmopelwa G (2014). Ethno-meteorology and scientific weather forecasting: Small farmers and scientists’ perspectives on climate variability in the Okavango Delta, Botswana. Clim Risk Manag.

[67] Boillat S, Berkes F (2013). Perception and Interpretation of Climate Change among Quechua Farmers of Bolivia: Indigenous Knowledge as a Resource for Adaptive Capacity. Ecol Soc.

[68] Hartter J, Stampone MD, Ryan SJ, Kirner K, Chapman CA, Goldman A (2012). Patterns and perceptions of climate change in a biodiversity conservation hotspot. PLoS One.

[69] Mercer J, Kelman I, Alfthan B, Kurvits T (2012). Ecosystem-based adaptation to climate change in caribbean small island developing states: Integrating local and external knowledge. Sustain.

