# Erratum to: Riverine fishers’ knowledge of extreme climatic events in the Brazilian Amazonia

**DOI:** 10.1186/s13002-017-0143-1

**Published:** 2017-04-24

**Authors:** Ana Isabel Camacho Guerreiro, Richard J. Ladle, Vandick da Silva Batista

**Affiliations:** 1 National Institute of Amazonian Research (INPA), Av. André Araújo, 2.936, Petrópolis. CEP 69.067.375, Manaus, AM Brazil; 2 Federal University of Alagoas (UFAL), Institute of Biological and Health Sciences (ICBS), Campus A. C. Simões; Av. Lourival Melo Mota s/n, Tabuleiro dos Martins. CEP 57.072.900, Maceió, AL Brazil; 3 School of Geography and the Environment, University of Oxford, Oxford, UK

## Erratum

In the original publication [1] figure 4 and 5 are been switched and do not correspond to each other.
